# Plant–pathogen interactions and ambient pH dynamics

**DOI:** 10.1007/s44154-024-00183-9

**Published:** 2025-01-03

**Authors:** Zhi Li, Yanchun Fan, Ronghui Wu, Min Gao, Xiping Wang

**Affiliations:** 1 State Key Laboratory for Crop Stress Resistance and High-Efficiency Production, College of Horticulture, Northwest A&F University, Yangling, Shaanxi 712100 China; 2 Key Laboratory of Horticultural Plant Biology and Germplasm Innovation in Northwest China, Ministry of Agriculture, Northwest A&F University, Yangling, Shaanxi 712100 China; 3 College of Forestry, Northwest A&F University, Yangling, 712100 Shaanxi China

**Keywords:** Ambient pH, Plant immunity, Stomatal closure, Acidification, Alkalinization

## Abstract

Ambient pH performs a crucial role in plant immunity, abiotic stress response, and plant growth and development. Plant–pathogen interactions influence a dynamic pH environment. Host acidification and alkalinization are under the regulating actions of acidophilic and alkaliphilic pathogens, respectively. Plasma membrane H^+^-ATPases regulate pH homeostasis, and their activity is modulated during pathogen-associated molecular pattern- (PTI) and effector-triggered immunities (ETI). Recent studies have provided insights into the interplay between pathogens and plants when controlling ambient pH. Here, we review pH dynamics in relation to plant immunity and focus on its relationships with PTI, ETI, stomatal immunity, programmed cell death, calcium ion (Ca^2+^), and reactive oxygen species. Comprehension of the complexity of ambient pH regulation may provide new strategies for plant disease control and sustainable agriculture.

**Supplementary Information:**

The online version contains supplementary material available at 10.1007/s44154-024-00183-9.

## Introduction

**Fig. 1:**
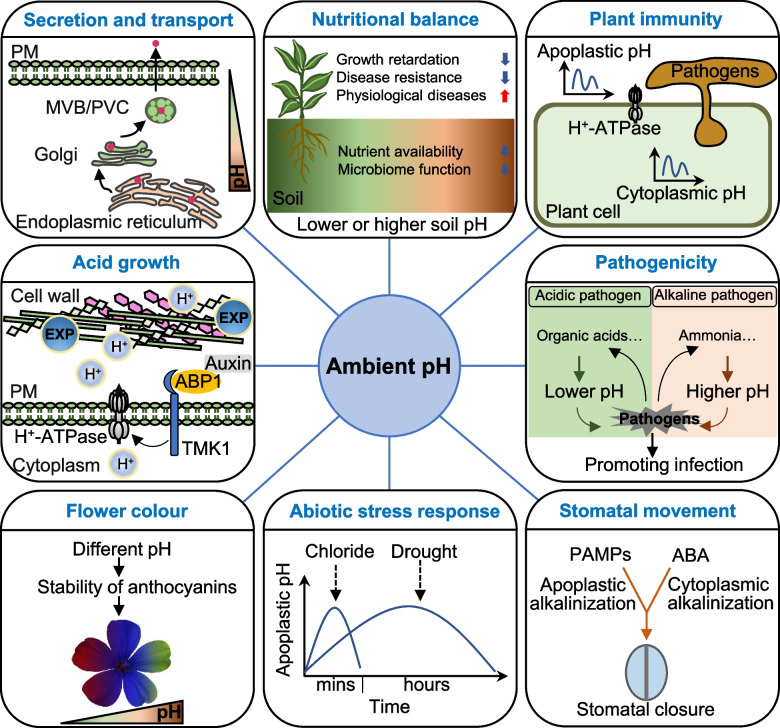
Ambient pH plays an important role in the biological processes of plants and pathogens. It affects the secretory pathway, growth, nutritional balance, immunity, abiotic stress response, and stomatal movement of plants and the success of pathogens. Intracellular substances are secreted from the endoplasmic reticulum to the *trans*-Golgi network (TGN) at neutral pH and then released at an acidic pH by the MVB/PVC compartment. Ambient pH also affects enzyme activity and transport during carbohydrate metabolism. ABP1 is an auxin receptor for the TMK1-based cell-surface signal. TMK1 phosphorylates and activates the plasma membrane (PM) H^+^-ATPase and is required for auxin-induced H.^+^-ATPase activation, apoplastic acidification, and cell expansion. The stability of anthocyanins depends on the environmental pH, thus affecting the color of flowers. In soil, excess ammonium concentrations lead to increased cell acidity and impairs growth. Improper soil pH often causes nutritional imbalance, disturbance of the soil microbial community, and various physiological diseases. Abiotic stress, such as drought and salt stress, can cause host apoplastic alkalinization, whereas biotic stress can induce apoplastic acidification or alkalization. Ambient pH can be disturbed by pathogens and the changed pH can also be perceived by the pathogen, which regulate the expression of related genes and enhance their own pathogenicity. In addition, stomatal closure is accompanied by apoplastic alkalinization and cytoplasmic alkalinization in response of PAMPs and ABA in guard cells, respectively. ABA, abscisic acid; ABP1, auxin-binding protein 1; MVB, multivesicular body; PAMPs, pathogen-associated molecular patterns; PM, plasma membrane; PVC, prevacuolar compartment; TMK1, transmembrane kinase 1

pH changes in plant tissues during various physiological processes signal the regulation of various aspects of growth, development, and environmental responses (Fig. 1). Ambient pH dynamics often involves in acidification or alkalinization in the apoplast, cytoplasm, and vacuole. Auxin-mediated apoplastic acidification contributes to the regulation of morphogenesis and cell expansion (Dang et al. 2020). Recent data indicate that auxin receptor auxin-binding protein 1 and transmembrane kinase 1 activate plasma membrane (PM) H^+^-ATPase through phosphorylation, which leads to cell wall acidification and promotes cell elongation and tissue growth (Friml et al. 2022; Lin et al. 2021). Cytoplasmic acidification correlates with development of root cap cells (Wang et al. 2023) and pollen tube during self‐incompatibility (Goring et al. 2023). Gradual acidification contributes to the secretory pathway of intracellular compartments (Shen et al. 2013). By contrast, transient apoplastic alkalinization can be attributed to various stresses, such as chloride salt and drought (Elmore and Coaker 2011; Geilfus 2017). Furthermore, pH dynamics has a crucial effect on several aspects of plant biology, including nutrient absorption, microbial diversity, peptide–receptor perception, and plant immunity (Han et al. 2023; Hartemink and Barrow 2023; Li et al. 2023).

**Fig. 2:**
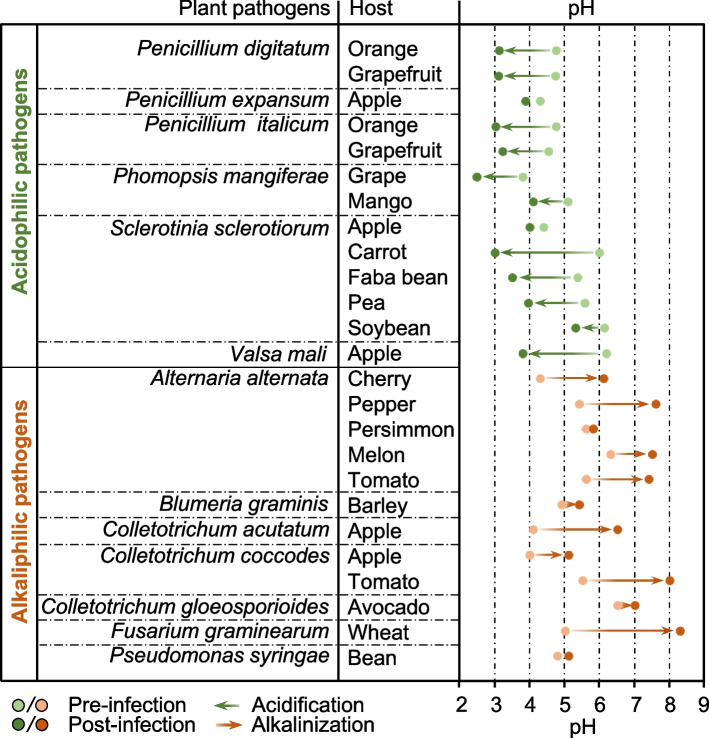
Apoplastic pH shifts in various host plants modulated by acidophilic and alkaliphilic pathogens. pH of plant tissues increases or decreases at different time points after inoculation compared with healthy tissues, which include leaves, fruits, roots, and twigs (Table S1 in Supplemental Information online). The green and orange–red arrows indicate acidification and alkalinization

The modulation of host pH is a common infection strategy for most plant pathogens to improve their pathogenicity and adaptability. During their coevolution with pathogens, plants evolved pathogen-associated molecular pattern (PAMP)- (PTI) and effector-triggered immunity (ETI) for the detection of invading pathogens and subsequently activation of defense mechanisms (Ngou et al. 2022). The PTI pathway features a transient extracellular alkalinization, and various types of ETI pathway exhibit an association with apoplastic acidification or alkalinization (Elmore and Coaker 2011). Although programmed cell death (PCD) facilitates plant immunity induction through cytoplasmic acidification, vacuolar acidification remains essential for plant antiviral immunity (Yang et al. 2022). In addition, pathogens have evolved the capacity to disrupt host pH to improve infection (Fig. 2). Pathogen-induced acidification and alkalinization occur during pathogen–host interactions involving fungi, bacteria, viruses, and aphids (Guo et al. 2023; O’Leary et al. 2016; Prusky and Yakoby 2003; Yang et al. 2022). The apoplastic acidification mediated by pathogens seems to achieve their infection goals by affecting cell wall growth and promoting degradation (Guo et al. 2023; Kesten et al. 2019), while apoplastic alkalization is critical for the formation of *Pseudomonas syringae*-induced lesions on leaves of *Phaseolus vulgaris* (Geilfus et al. 2020).

PM H^+^-ATPases regulate intracellular and extracellular pH homeostasis and play key roles in cell physiology and plant immunity (Falhof et al. 2016; Miao et al. 2022). Acidification or alkalinization occurs through the regulation of PM H^+^-ATPases during plant/pathogen interactions. This review focuses on recent advances linking pH dynamics and plant immunity and summarize recent findings related to the mechanisms of acidization and alkalinization associated with pathogens and the relationship of pH dynamics with PTI, stomatal immunity, ETI, PCD, Ca^2+^, and reactive oxygen species (ROS). Insights into pH dynamics may applied for the improvement plant disease resistance.

## Apoplastic acidification

**Fig. 3:**
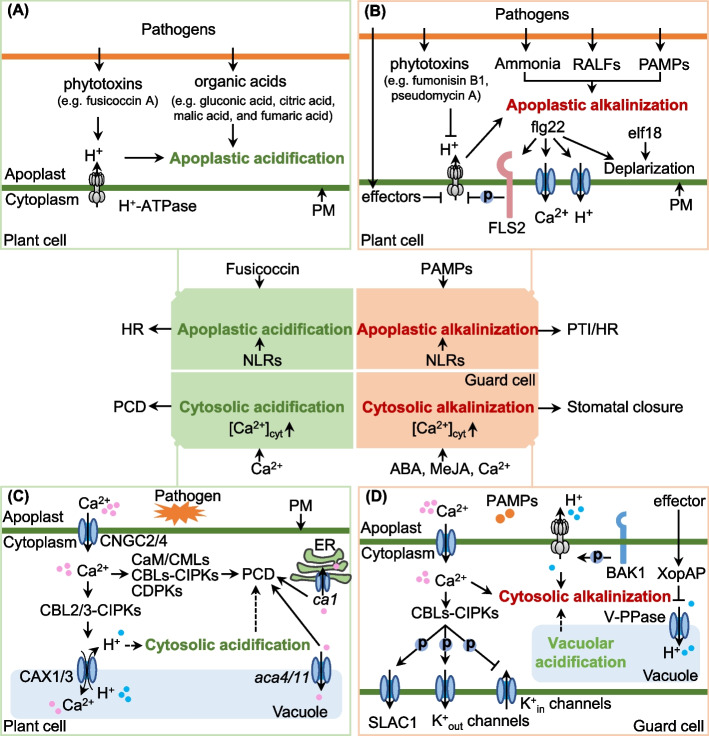
Acidification and alkalinization influenced by pathogens and signal molecules. **A** Main pathway of apoplastic acidification involving the release of organic acids, which results from organic acid metabolism in fungal cells. Phytotoxins from pathogens also activate the PM H^+^-ATPase activities of the host. **B** Regulation of apoplastic alkalinization through the release of ammonia, PAMPs, RALFs, effectors, and phytotoxins from pathogens. Ammonia release can be attributed to the uptake of amino acids and improvement of amino acid metabolism in fungal cells and the increase in ambient pH. The secretion of chitin and sterols from fungi is another cause of host alkalinization. **C** Cytoplasmic Ca^2+^ signals show a close relation to PCD and cytoplasmic acidification. Although apoplastic and cytoplasmic acidification can induce HR or PCD, cytoplasmic acidification is more dependent on [Ca^2+^]_cyt_. **D** During stomatal closure, the regulation of cytoplasmic alkalinization and vacuolar acidification is influenced by ABA, [Ca^2+^]_cyt_, and effectors in guard cells. ABA, abscisic acid; BAK1, BRI1-associated receptor kinase 1; FLS2, the leucine-rich repeat receptor kinases flagellin-sensitive 2; PAMPs, pathogen-associated molecular patterns; PM, plasma membrane; RALF, rapid alkalinizing factor

Host apoplastic acidification is attributed to the release of organic acids from acidophilic pathogens and the activation of plant PM H^+^-ATPases (Fig. 3A). During host acidification, oxalic acid, citric acid, malic acid, and succinic acid are secreted by *Botrytis cinerea* and *Sclerotinia sclerotiorum* (Billon-Grand et al. 2012), and oxalic acid and citric acid are secreted by *Aspergillus niger* (Ruijter et al. 1999). *Phomopsis mangiferae* acidifies the host by secreting gluconic acid, citric acid, malic acid, and fumaric acid (Davidzon et al. 2010), whereas citric acid, fumaric acid, and oxalic acid are secreted by *Penicillium* spp. (Prusky et al. 2004). Recently, secreted fusicoccin A from *Neofusicoccum parvum* combines 14‐3‐3 proteins and triggers extracellular acidification via binding to PM H^+^-ATPases (Khattab et al. 2023). Moreover, low pH growth condition has also been shown to activate H^+^-ATPase activities in *Oenococcus oeni* (Fortier et al. 2003), *Penicillium simplicissimun* (Burgstaller et al. 1997), and *Saccharomyces cerevisiae* (Carmelo et al. 1996) as well as in several plant species (Yan et al. 1998; Zhu et al. 2009). Apoplastic acidification of *Arabidopsis thaliana* roots is a result of the activation of the root proton pump through phosphorylation in response to the fungus *Fusarium oxysporum* (Kesten et al. 2019). Further genetic analyses have shown that *B*. *cinerea* MAPK kinase (BcMkk1) negatively regulates oxalic acid biosynthesis via impeding phosphorylation of Per-Arnt-Sim (PAS) kinase BcRim15 by the Ser/Thr kinase BcSch9 (Yin et al. 2018). Host tissue acidification and organic acids secretion are regulated by VELVET complex in *B*. *cinerea* and PM H^+^-ATPases (Pma1) in *Valsa mali* (Müller et al. 2018; Zhang et al. 2023a). For aphid pathogens, the secretion of carbonic anhydrase II (CA-II) by the aphid *Myzus persicae* results in apoplastic acidification of tobacco leaves (Guo et al. 2023). However, how the host perceives acidification signals for defense responses, such as whether it enhances the secretion of alkaline substances, remains unclear.

## Apoplastic alkalinization

Over the past few decades, extracellular pH alkalinization has been regarded as one of the markers of plant immune response, especially in the PTI pathway. In addition to PMAPs induction, apoplastic alkalinization has been associated with the release of rapid alkalinizing factor (RALF), ammonia, chitin, sterols from the pathogens, and the inhibition of plant PM H^+^-ATPases by secreted metabolites (Fig. 3B).

### PAMPs triggered alkalinization

**Fig. 4:**
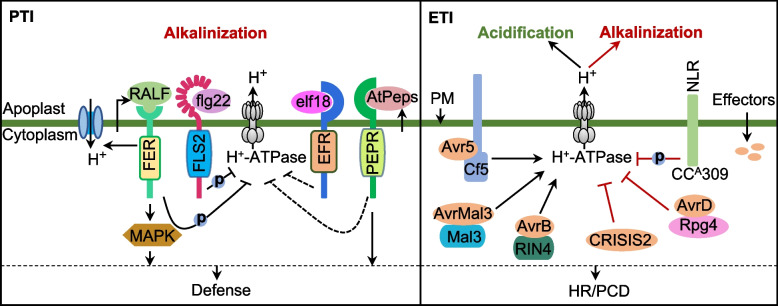
Apoplastic pH shift modulated by PTI and ETI. Changes in ambient pH depend on PM H^+^-ATPase activity, which is regulated by host recognition of PAMPs and effectors from pathogens and plant peptides (peps and RALFs). The alkalinization induced by flg22 depends on the phosphorylation and inhibition of PM H^+^-ATPase activity by its receptor FLAGELLIN SENSITIVE2, whereas the elf18-induced mechanism of extracellular alkalinization remains unclear. RALF-induced alkalinization involves its receptor kinase FERONIA (FER), which phosphorylates and inhibits PM H^+^-ATPase activity. In addition, the involvement of RALF in PTI signal translation depends on the downstream mitogen-activated protein kinase pathway. ETI includes convergent pathways related to ambient pH. Apoplastic acidification or alkalinization depends on the activation or inhibition of PM H^+^-ATPase during ETI, respectively. Arrows represent promotion. Black ended arrows represent suppression. Solid lines represent a verified relationship, and dotted lines represent connections that need to be characterized further. P in the blue circle indicates phosphorylation. FLS2, FLAGELLIN-SENSING 2; HR, hypersensitive response; NLR, nucleotide-binding and leucine rich repeat protein; PCD, programmed cell death; PM, plasma membrane; RALF, rapid alkalinizing factor

Apoplastic alkalinization is a result of PM depolarization, ion fluxes, and the inhibition of PM H^+^-ATPases upon perception of PAMPs (Fig. 4). The alkalinization as an early cellular response was found in response to *PAMPs*, such as flagellin 22 (flg22), elongation factor-Tu (elf18 and elf26), 13-amino acid peptide (pep13), lipopolysaccharides (LPS), and chitin (Albus et al. 2001; Felix et al. 1993; Jeworutzki et al. 2010; Kunze et al. 2004; Nurnberger et al. 1994), but not in response to nlp20 and elf12 (Kunze et al. 2004; Bohm et al. 2014). PM depolarization induced by elf18 and flg22, and flg22-dependemt PM depolarization occurs through the involvement of the FLS2 receptor with calcium-activated anion channels (Jeworutzki et al. 2010). The application of PAMPs (flg22 and pep13) also increases the H^+^/Ca^2+^ influxes and the Cl^−^ /K^+^ effluxes (Jeworutzki et al. 2010; Nurnberger et al. 1994). The inhibition of PM H^+^-ATPase by flg22 may depend on its receptor kinase FLAGELLIN-SENSING 2 (FLS2), which phosphorylates the PM H-ATPase at Ser-899, causes pump inactivation and apoplastic alkalinization (Nuhse et al. 2007). Additionally, plant elicitor peptides (peps) are perceived by their receptors (PEPRs) to cause medium alkalinization, and pep–PEPR interaction promotes immune responses (Yamaguchi et al. 2006, 2010). Alkalinization in turn promotes the Pep1-PEPRs binding and enhances root immunity in Arabidopsis (Liu et al. 2022b). Chitin fragments and chitosan are also potent PAMPs and elicit PTI in many plant species. Interestingly, chitohexaose NAG6-induced medium alkalinization was shown to be strongly suppressed by the chitinase-like effector (MpChi) of *Moniliophthora perniciosa* in tobacco cells (Fiorin et al. 2018). This suggested that pathogens may deploy effectors to disturb the alkalinization signal during PTI. It has been determined that transient alkalinization of the apoplast may be correlated with the degree of resistance toward pathogens, and an important next step will be to elucidate how plants transmit and trigger immune responses involving alkalinization signals.

### RALFs mediated alkalinization

RALFs induce the rapid alkalinization of the extracellular compartment and regulate growth, development, and immunity in plant (Zhang et al. 2020b). However, some fungi and nematodes also produce RALFs, cysteine-rich peptides that effectively trigger the alkalinization of plant tissues. *F. oxysporum* has been shown to secrete F-RALF and RALF-B peptides that trigger rapid extracellular alkalinization in tomato and tobacco (*Nicotiana tabacum*) (Masachis et al. 2016; Thynne et al. 2017), and similar results were reported in plant-nematode interactions, where MiRALF1 and MiRALF3 from the nematode (*Meloidogyne incognita*) rapidly alkalized the extracellular medium and plant roots (Zhang et al. 2020a). Although the mechanism by which pathogenic RALFs affect host H^+^-ATPase activity is not clear, plant RALFs-triggered alkalinization are known to inhibit H^+^-ATPase activity and induce H^+^ influx (Gjetting et al. 2020; Li et al. 2022b). It has been shown that the interaction of RALF with FERONIA (FER), a major RALF peptide receptor, results in the phosphorylation of Ser899 of the PM H^+^-ATPase, thereby leading to pump inactivation (Haruta et al. 2014). RALF peptides may, however, function via interactions with many different downstream partners, such as cell-wall-associated leucine-rich repeat (LRR) extensin (LRX), LORELEI (LRE)-like glycosylphosphatidylinositol (GPI)-anchored proteins (LLGs), and mitogen activated protein kinase (MAPK) cascades in plants (He et al. 2023; Ortiz-Morea et al. 2022; Zhang et al. 2023c). Currently, some nematode RALFs can bind to the extracellular domain of plant FER (Zhang et al. 2020a), but it is still unclear whether the RALF- FER signaling can affect the H^+^-ATPase activity. The depth mechanism of extracellular alkalinization induced by RALF from pathogens still needs a long way to go.

### Secreted metabolites triggered alkalinization

An increase in extracellular pH is accompanied with ammonia accumulation and secretion from alkaliphilic pathogens attack (Fernandes et al. 2017). It requires the uptake of amino acids, the degradation of nitrogen sources, and nutrient deprivation, and the accumulation of ammonia results in the activation of fungal appressorium formation (Miramon and Lorenz 2016; Shnaiderman et al. 2013). In addition to ammonium, induction of extracellular alkalinization is also triggered by pathogen chitin fragments in tomato and sterols in *Beta vulgaris* (Felix et al. 1993; Rossard et al. 2010), and syringolide 1, an elicitor secreted by *P*. *syringae* into soybean (*Glycine max*) callus cells (Atkinson et al. 1996). A recent study found that secreted proteins and one of its components exo-polygalacturonase (PehC) from *Ralstonia solanacearum* trigger extracellular alkalinization in tomato roots (Ke et al. 2023). Furthermore, extracellular alkalinization can also result from the inhibition of H^+^-ATPase activity, mediated by pathogen phytotoxins or metabolites (Havshoi and Fuglsang 2022), such as fumonisin B1 and fusaric acid from *Fusarium* spp. (Gutierrez-Najera et al. 2005; Wang et al. 2014), pseudomycin A from *P*. *syringae* (Di Giorgio et al. 1997), and tenuazonic acid from *Stemphylium loti* (Bjork et al. 2020).

## pH dynamics and stomatal closure

Stomatal closure is under modulation by PAMPs in biotic pathways and abscisic acid (ABA) in abiotic pathways, which are associated with pH dynamics. This regulation relies on the activation or inactivation of PM H^+^-ATPase, vacuolar proton pump, and membrane potential. Stomatal closure is a crucial part of PTI and limits pathogen entry, whereas rapid apoplastic alkalinization is a key and early step that result in stomatal closure upon PAMP perception (Arnaud et al. 2023b; Zou et al. 2021). flg22 treatment caused no significant change in [H^+^]_cyt_ in guard cells (Li et al. 2021). However, early stomatal closure has been characterized by cytoplasmic alkalinization in response to ABA and methyl jasmonate (MeJA) (Suhita et al. 2004). PM H^+^-ATPase activity performs a specific function in an ABA-dependent pathway controlling stomatal closure (Merlot et al. 2007). The function of ABA involves the activation of BRI1-associated receptor kinase 1 to phosphorylate *A. thaliana* PLASMA MEMBRANE PROTON ATPASE2 at Ser-944 and activate the H^+^-ATPase activity; which this condition results cytoplasmic alkalinization and initiates stomatal closure followed by Ca^2+^ elevation (Pei et al. 2022). The stomatal closure induced by ABA occur in Ca^2+^-independent and -dependent manners. Sucrose nonfermenting 1-related protein kinase 2.6 (also known as open stomata 1) not only phosphorylates and activates SLOW ANION CHANNEL-ASSOCIATED1 (SLAC1) in a Ca^2+^-independent manner but also activates four cyclic nucleotide-gated channels (CNGC5/6/9/12) to trigger [Ca^2+^]_cyt_ signals that consequently activate SLAC1 and SLAC1 HOMOLOGUE 3 in a Ca^2+^-dependent manner (Fig. S1, Geiger et al. 2009; Yang et al. 2024). The treatment with MeJA increased PM H^+^-ATPase activity in the root tips of lettuce (*Lactuca sativa*) seedlings (Zhu et al. 2015); however, no concrete evidence has been provided for such regulation in guard cells.

Regardless of their differences, PAMPs- and ABA-induced alkalinization showed similarities in terms of vacuolar acidification in response to flg22 and ABA in guard cells (Li et al. 2024). The vacuolar proton ATPase (V-PPase)-mediated vacuolar acidification is necessary for the rapid ABA-induced stomatal closure (Bak et al. 2013). Notably, the effector XopAP from *Xanthomonas oryzae* pv. *oryzicola* inhibits vacuolar acidification, which leads to stomatal opening and infection (Liu et al. 2022a). These results imply the linkage of cytoplasmic alkalinization to vacuolar acidification during signal transduction of stomatal closure in guard cells (Fig. 3D, Zhang et al. 2001). Another similarity is that PAMPs and ABA can cause PM depolarization, which leads to stomatal closure (Jeworutzki et al. 2010; Meimoun et al. 2009). PM depolarization promotes stomatal closure, whereas hyperpolarization exhibits a relation to stomatal opening. In addition, stomatal closure may involve PAMPs and ABA partially sharing pH-dependent signaling pathways. Butyric acid (BTA) is a weak acid commonly used to induce moderate cytosolic acidification. This compound induces cellular acidification and can inhibit flg22- and ABA-induced stomatal closure (Li et al. 2024; Pei et al. 2022).

Cytoplasmic alkalinization causes the activation of K^+^ efflux and inactivation of K^+^ influx, which modulate ion mobilization in *Vicia sativa* guard cells (Blatt and Armstrong 1993). By contrast, guard cell acidification activates K^+^ channel in *A. thaliana* 1 (KAT1) and KAT1 homolog of potato (KST1) and reduces the activity of the guard cell outward rectifying K^+^ channel (GORK) (Ache et al. 2000). Thus, apoplastic alkalinization maybe closely related to PAMPs, whereas cytoplasmic alkalinization may be involved with ABA in guard cells. PAMPs and ABA largely contribute to the inducement of stomatal closure via membrane depolarization and vacuolar acidification. However, these molecules may differ in the regulation of pH-dependent stomatal closure.

## pH dynamics and ETI

Compared with apoplastic alkalinization during PTI, the types of pathogens and the *R* genes determine acidification or alkalinization during ETI (Fig. 4). Apoplastic acidification during ETI depends on the activation of PM H^+^-ATPases for an incompatible plant–pathogen interaction. For example, the *Cladosporium fulvum* effector Avr5 is recognized by the R protein Cf5 in tomato cells, leading to the activation of the PM H^+^-ATPase and extracellular acidification (Vera-Estrella et al. 1994). Similarly, apoplastic acidification resulting from activated PM H^+^-ATPase is also observed during the interaction between barley Mla3 and the AvrMla3 from powdery mildew (*B. graminis*) (Zhou et al. 2000). The *P*. *syringae* effector AvrB targets the plant immune regulator RIN4, and AvrB and RIN4 enhance plant PM H^+^-ATPase activity, while AvrB enhances bacterial virulence in a RIN4-dependent manner through inducing stomatal opening mediated by jasmonate (JA) signaling in guard cells (Zhou et al. 2015; Lee et al. 2015). Apoplastic alkalinization and HR mediated by different *R* genes have also been linked to incompatible interactions between barley (*Hordeum vulgare*) and *B. graminis* (Felle et al. 2004). Similar results were found during the interaction of *P*. *syringae* avrD and soybean Rpg4*,* confirmed by the absence of HR in two *rpg4* cultivars (Atkinson et al. 1996). A recent study found that PM-localized NLRs with CC domains CC^A^309, a nucleotide-binding and leucine rich repeat proteins, inhibits PM H^+^-ATPase activity, leading to extracellular alkalinization, followed by PCD (Lee et al. 2022). Additionally, a RxLR effector CRISIS2 from *Phytophthora capsici* also induces apoplastic alkalinization by inhibiting PM H^+^-ATPase activity (Seo et al. 2023). Thus, the apoplastic alkalinization or acidification may regulate the pH-dependent self-activation and protein fold of plant resistance proteins, coreceptor of resistance proteins, or pathogen effectors (Paulus et al. 2020; Dawson et al. 2009).

## pH dynamics and PCD

PM H^+^-ATPase activity links defense responses and PCD in plants. PCD occurs during development or under biotic and abiotic stresses. Cytoplasmic acidification exhibits a correlation with developmentally controlled PCD (dPCD) in root cap cells (Wang et al. 2023) and pollen tube during self‐incompatibility response within a few minutes (Kong et al. 2021; Wang et al. 2022b) and pathogen-triggered PCD (pPCD) (Huysmans et al. 2017; Atkinson et al. 1985). This correlation has also been observed in animal cells and yeast cells (Basu and Haswell 2020; Niu and Spradling 2022). Accumulated pieces of evidence indicate the linkage of nucleotide-binding leucine-rich repeat proteins (NLRs) and effector-induced cell death to the inhibition of PM H^+^-ATPase activity (Lee et al. 2022; Seo et al. 2023). The PM H^+^-ATPase activator fusicoccin compromise several NLR-induced cell deaths (Seo et al. 2023). By contrast, the overexpression of bacterial H^+^-ATPase in tobacco plants triggers a hypersensitive response (HR)-like PCD (Mittler et al. 1995). In addition, a low-pH buffer and PM H^+^-ATPase activation increase the number of cells exhibiting HR (Zhou et al. 2000). Moreover, pathogens secrete organic acids that not only cause extracellular acidification but also induce cell death (Jiao et al. 2022; Kim et al. 2008); this finding suggests the promotion of pPCD is intensified by the activation of PM H^+^-ATPase and extracellular acidification. Such a contradiction may be attributed to the disturbance of PM potential by PM H^+^-ATPase and pH shift, which lead to an imbalance in ion fluxes during pPCD (Salguero-Linares and Coll 2023). The PM potential in healthy cells is coupled with the regulation of ion channels and transporters; however, the loss of PM potential shows an association with HR-like death (Liu et al. 2007; Pike et al. 2005).

Among various ion fluxes, cytoplasmic Ca^2+^ signals bear a close relation to pPCD. In the presence of flg22, Ca^2+^ efflux increases the level of external Ca^2+^; then, Ca^2+^ reaches the cytoplasm through cyclic nucleotide gated channels (CNGCs), which elevates the level [Ca^2+^]_cyt_ sensed by calcineurin B-likes (CBL) 2/3 and CBL-interacting protein kinases (CIPK) 3/9/36. The subsequent phosphorylation and activation of vacuolar Ca^2+^/H^+^ exchangers (CAX1/3) under the action of these Ca^2+^-sensor kinases enable the transport of excess Ca^2+^ to the vacuole and release of H^+^ into the cytoplasm (Wang et al. 2024). CCHA1 is a chloroplast-localized potential Ca^2+^/H^+^ antiporter in *A*. *thaliana*, and cytoplasmic pH in *ccha1* mutant is higher than in the wild-type (Wang et al. 2016). The findings suggest the possible relation of cytoplasmic acidification to Ca^2+^-dependent CAX activation (Fig. 3C). The shape of cytoplasmic Ca^2+^ signal depends on the balance among CNGC-mediated Ca^2+^ influx, PM-, endoplasmic reticulum (ER)- mediated efflux, and tonoplast-localized Ca^2+^ pumps (Köster et al. 2022). CNGC2 and CNGC4 contribute to the induction of HR (Jurkowski et al. 2004; Clough et al. 2000). Knockout of vacuolar Ca^2+^ pumps *ACA4* and *ACA11* reveal salicylic acid (SA)-dependent PCD in Arabidopsis leaves during pathogen attack (Boursiac et al. 2010). Silencing of ER-localized Ca^2+^-ATPase (*NbCA1*) accelerates the PCD induced by pathogens (Zhu et al. 2010). These data imply that pPCD is an outcome of [Ca^2+^]_cyt_ elevation, Ca^2+^ sensing, and Ca^2+^ signal transduction (Ren et al. 2021). Furthermore, pPCD may be linked to the nuclear Ca^2+^ ([Ca^2+^]_nuc_), which is generated by Ca^2+^ influx from the cytoplasm (Jiang and Ding 2023; Zhu et al. 2010). Ca^2+^-dependent nuclease has been shown to participate in nuclear DNA degradation during dPCD in *Citrus* fruits (Liang et al. 2024) but not pPCD. Nuclear-membrane localized CNGCs (CNGC15a/b/c) and ER-localized Ca^2+^-ATPase (NbCA1) account for nuclear calcium oscillation; their actions cause alterations in the chromatin state and regulates transcriptional reprogramming (Charpentier et al. 2016; Jiang and Ding 2023; Zhu et al. 2010). The relationship between [Ca^2+^]_nuc_ signature and pPCD still needs further investigation.

## pH dynamics and Ca^2+^

Cell types and signaling molecules determine the type of relationship between pH dynamics and [Ca^2+^]_cyt_. Upon flg22, stomatal closure occurs with apoplastic alkalinization and [Ca^2+^]_cyt_ elevation; however, [H^+^]_cyt_ in guard cells showed no significant change (Arnaud et al. 2023b; Li et al. 2021). By contrast, flg22 functions in the inducement of [Ca^2+^]_cyt_ elevation and cytosolic acidification in mesophyll cells (Li et al. 2021). The stomatal closure in guard cells induced by ABA is accompanied with rapid cytosolic alkalinization followed by [Ca^2+^]_cyt_ elevation (Irving et al. 1992; Li et al. 2021; Pei et al. 2022). In leaf and root cells, increases in [Ca^2+^]_cyt_ are accompanied with cytosolic acidification in response to external stimuli (Behera et al. 2018). BTA induces cytosolic acidification, which reduces the [Ca^2+^]_cyt_ levels in guard cells, whereas BTA potentiates [Ca^2+^]_cyt_ in mesophyll cells (Li et al. 2024). The difference among various cell types can be attributed to the pH sensitivity of Ca^2+^ channels. Extracellular K^+^ and anion fluxes affect cellular Ca^2+^ and H^+^, which cause shifts in cytosolic and vacuolar pH and changes in [Ca^2+^]_cyt_ and membrane potential (Li et al. 2024). In addition, extracellular Ca^2+^ closes stomata, causes transient oscillatory cytosolic alkalinization, and increases [Ca^2+^]_cyt_ in guard cells (Li et al. 2024; Gilroy et al. 1991). However, organic acids from pathogens, such as oxalic acid and citric acid, can chelate Ca^2+^ and reduce its activity (Cessna et al. 2000; Jiao et al. 2022). The chelation of extracellular Ca^2+^ results in the reduced amplitude of [Ca^2+^]_cyt_ elevation and cytosolic acidification in root tip cells (Behera et al. 2018). Therefore, these findings suggest that various signaling molecules that affect [Ca^2+^]_cyt_ and [H^+^]_cyt_ dynamics are linked to cell types, ion channels, and membrane potential.

During pathogen infection, [Ca^2+^]_cyt_ performs certain functions in PTI and ETI (Jiang and Ding 2023). However, the connection of [Ca^2+^]_cyt_ and pH dynamics in both types of immunity remains incompletely understood. ABA- and PAMP-induced stomatal closures lead to an increase in [Ca^2+^]_cyt_. Regardless, further exploration is needed to determine whether their shared pH-dependent mechanisms trigger stomatal closure. In addition to guard cells, [Ca^2+^]_cyt_ elevation shows a close association with cytosolic acidification during pPCD and dPCD (Goring et al. 2023; Huysmans et al. 2017). Thus, pPCD and dPCD may occur beyond a certain threshold of [Ca^2+^]_cyt_.

## pH dynamics and ROS

Alkalinization has been linked to ROS burst in host cells during PTI. ROS elevation occurred in tomato and *N*. *benthamiana* leaves induced by *F*. *oxysporum* rapid alkalinizing factor (RALF)-B (Thynne et al. 2017) and in tomato roots induced by PehC from *Ralstonia solanacearum* during extracellular alkalinization (Ke et al. 2023). Apoplastic ROS production depends on nicotinamide adenine dinucleotide phosphate oxidases (NOXs or respiratory burst oxidase homologs (RBOHs), cell wall peroxidases (PRXs), and amine oxidases (Castro et al. 2021). Extracellular alkalinization is a prerequisite for PRX-mediated extracellular ROS production (Bolwell et al. 2002). Furthermore, the activation of PM H^+^-ATPase causes reduction in the ROS burst in response to flg22 (Keinath et al. 2010); thus alkalinization may be a crucial component of ROS signaling. Interestingly, RBOHF also participates in PAMP-induced apoplastic alkalinization in Arabidopsis guard cells (Arnaud et al. 2023b). Cytoplasmic alkalinization in guard cells precedes ROS production during MeJA- and ABA-induced stomatal closures (Pei et al. 2022; Suhita et al. 2004); this finding indicates the presence of a mutually reinforcing relationship between alkalinization and ROS production.

Extracellular signals also influence pH dynamics and ROS. The application of 20 and 200 µM H_2_O_2_ strengthened cytosol acidification within 10–20 min in guard cells, but stomatal aperture was unaltered in *Nicotiana tabacum* (Li et al. 2021). However, recent data show that the application of 10 and 100 µM H_2_O_2_ effectively induced stomatal closure in Arabidopsis (Arnaud et al. 2023b). Such a contradiction in the results may be attributed to the sensitivity of various plants to H_2_O_2_. Conversely, acidic extracellular pH suppressed the flg22-triggered cytoplasmic ROS burst in Arabidopsis seedlings (Yu et al. 2019). Therefore, the importance of cytoplasmic or apoplastic ROS for stomatal closure should be explored in detail.

ROS elevation in cytoplasm is closely associated with cytoplasmic acidification during dPCD (Goring et al. 2023). Chloroplastic ROS contribute to ETI-induced pPCD in response to pathogens; cytoplasmic acidification also occurs during pPCD (Atkinson et al. 1985; Liu et al. 2007), but knowledge on its correlation with the pPCD process compared with dPCD is limited. Thus, further studies are required to determine whether cytoplasmic acidification triggers the accumulation of cytoplasmic ROS during pPCD. Although the accumulation of cytoplasmic ROS partially depends on apoplastic ROS transport through aquaporin PIP2;1, cytoplasmic ROS are largely generated as metabolic by-products from chloroplasts and mitochondria (Waszczak et al. 2018). An increasing number of reports indicate that cytosolic oxidation is independent of RBOHD-dependent apoplastic oxidation events for stomatal immunity (Arnaud et al. 2023a, b). In addition, no clear distinction has been observed between intracellular and extracellular ROS or the various ROS types in numerous experimental systems. The relationship between pH dynamics and ROS requires further exploration through the integration of ROS compartmentalization (Castro et al. 2021) and accurate measurement of the types of ROS (Murphy et al. 2022).

## Concluding remarks

The perception and response to pH shifts is crucial for plant survival under biotic and abiotic stresses. In response to apoplastic acidification induced by aphids, plants expedited vesicle trafficking to augment pectin transportation and fortify the cell wall (Guo et al. 2023). Under flooding conditions, SLAH3 S-type anion channels serve as intracellular pH sensors that perceive cytosolic acidification and promote membrane depolarization in *A. thaliana* roots (Lehmann et al. 2021). Under alkaline stress, alkaline tolerance 1 negatively modulates the phosphorylation level of PIP2s, which reduces their H_2_O_2_ export activity under alkaline stress. This condition leads to the overaccumulation of H_2_O_2_ and reduced tolerance of crops to alkaline stress (Zhang et al. 2023b). However, whether a similar mechanism exists in pathogen-mediated alkalinization remains unclear. As extracellular pH sensors, peptides (root meristem growth factor 1 and Pep1) and their receptors (RGFR and PEPR) regulate extracellular pH-mediated growth and immunity in *A. thaliana* roots (Liu et al. 2022b). Regardless of the increase in the number of identified pH sensors, other cell type-specific pH sensors that sense pH shifts mediated by pathogens or abiotic stresses must be discovered. Such goals may be achieved with the assistance of large-scale and high-resolution single-cell multiomics analysis combined with live measurement of pH in specific cell types (Flynn et al. 2023; Shen and Zhao 2023).

The shift in pH varies between compatible and incompatible cultivars. Activation of PM H^+^-ATPase by *Sclerospora graminicola* infection occurs in all resistant cultivars of pearl millet but not in susceptible cultivars (Madhu et al. 2001); this condition suggests the correlation of the level of H^+^-ATPase with the degree of resistance. Conversely, the interaction of the alkaline pathogen *P*. *syringae* with *Phaseolus vulgaris* resulted in the higher degree of alkalinization caused by incompatible pathogens than that caused by compatible pathogens (O’Leary et al. 2016). Thus, the degree of alkalinization relates to microbial pathogenicity and defense response of the host. Further, scholars should focus on cultivar-specific differences in pH perception and regulation of ambient pH.

Given the acid-producing characteristic of pathogens, pH-sensitive fungicides can be used for the precise control of plant diseases based on pH-responsive delivery systems (Liang et al. 2021; Zhang et al. 2022). PM H^+^-ATPase, RALFs, and pH signaling transcription factor *PacC* not only modulate the ambient pH of the host but also regulate pathogen development and pathogenicity (Wang et al. 2022a; Wu et al. 2022). Therefore, plant disease resistance may be improved by silencing these genes via host- or spray-induced gene silencing (Li et al. 2022a; Lopez-Gomollon and Baulcombe 2022; Wang and Jin 2017). The successfully designed pH-responsive proteins can be assembled and depolymerized based on pH shifts (Shen et al. 2024). The release of disease-resistant proteins that target specific pH environments will contribute to disease prevention and control. In summary, pH dynamics reflect an ongoing evolutionary interaction linked to various aspects of plant pathosystems. Despite the recent advances and crucial and new insights, significant gaps in our knowledge remain. Answering these questions will contribute to the development of innovative strategies for the enhancement of plant immunity and disease resistance.

## Supplementary Information

Supplementary Material 1.

## Data Availability

Not applicable.

## Abbreviations

ABA: Abscisic acid
BTA: Butyric acid
dPCD: Developmentally controlled PCD
ETI: Effector-triggered immunity
FLS2: FLAGELLIN-SENSING 2
HR: Hypersensitive response
MeJA: Methyl jasmonate
PAMPs: Pathogen-associated molecular patterns
PCD: Programmed cell death
pPCD: Pathogen-triggered PCD
PRRs: Pattern recognition receptors
PTI: Pattern−triggered immunity
RALF: Rapid alkalinization factor
ROS: Reactive oxygen species
