# Supplementary material for: Plant–pathogen interactions and ambient pH dynamics
Source: Stress Biol. 2025 Jan 3;5(1):2. doi: 10.1007/s44154-024-00183-9 (PMC11695569; doi:10.1007/s44154-024-00183-9)
Supplement: Supplementary file 1 — Supplementary Material 1. [file 44154_2024_183_MOESM1_ESM.docx]

*Supplemental information*

**Plant–pathogen interactions and ambient pH dynamics**

Zhi Li^1,2^, Yanchun Fan^3^, Ronghui Wu^1,2^, Min Gao^1,2^, Xiping Wang^1,2*^

^1^State Key Laboratory for Crop Stress Resistance and High-Efficiency Production, College of Horticulture, Northwest A&F University, Yangling, Shaanxi 712100, China

^2^Key Laboratory of Horticultural Plant Biology and Germplasm Innovation in Northwest China, Ministry of Agriculture, Northwest A&F University, Yangling, Shaanxi 712100, China

^3^College of Forestry, Northwest A&F University, Yangling, Shaanxi 712100, China

*Correspondence: Xiping Wang (wangxiping@nwsuaf.edu.cn)


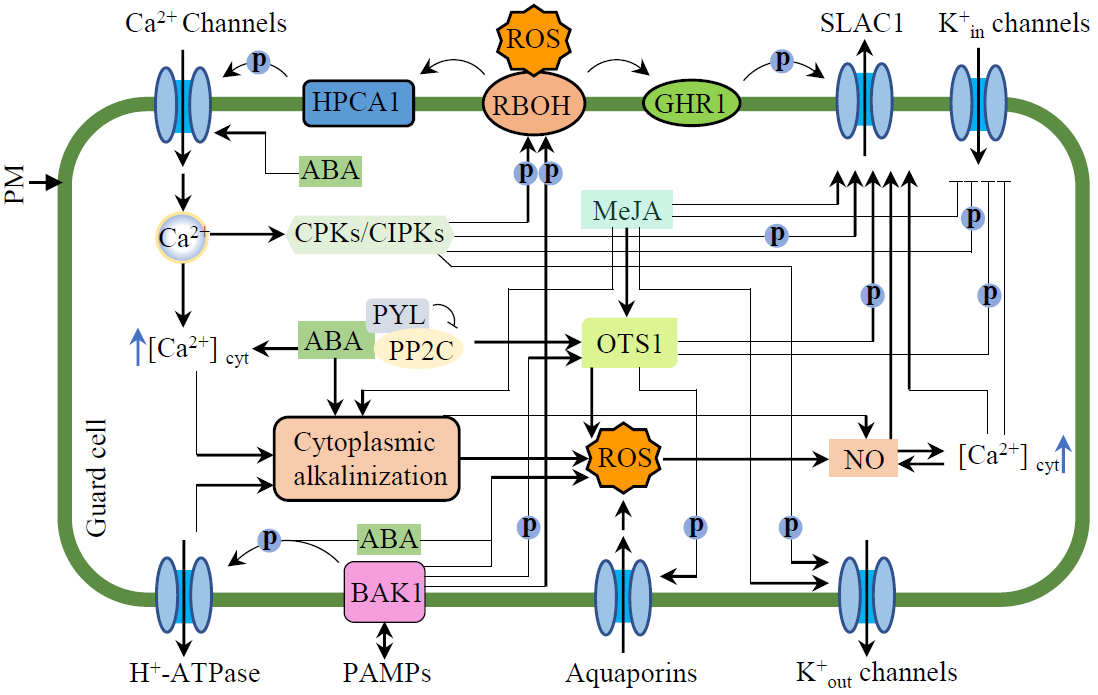


**Fig. S1** Guard cell signal transduction and stomatal closure regulated by cytoplasmic alkalinization. Stomatal closure can be manipulated by secondary messengers (ABA, MeJA, and NO), the activity of ion channels (K^+^, H^+^, and Ca^2+^ channels), ROS, ambient pH, and crosstalk of some of these components. A rise in ABA content in guard cells can be induced by pathogens. In the presence of ABA, BAK1 phosphorylates and activates PM H^+^-ATPase, produces ROS by activating RBOH NADPH oxidases, and activates Ca^2+^ influx. MeJA activates SLAC1 and K^+^_out_ channels and inhibits K^+^_in_ channels. NO production is induced by ABA and MeJA. NO elevation raises the concentration of cytosolic free Ca^2+^ ([Ca^2+^]_cyt_), through the upregulation of cyclic guanosine monophosphate (cADPR) and cyclic guanosine monophosphate (cGMP) and activates SLAC1 channels. Sucrose non-fermenting 1-related protein kinase 2.6 (also known as OST1, Open stomata 1) is regulated by ABA, MeJA, and the phosphorylation of BAK1. OST1 phosphorylates SLAC1 and PIP2;1 to activates SLAC1 channels and aquaporins but phosphorylates KAT1 to suppress K^+^_in_ channels. Cytoplasmic alkalinization results from the activation of PM the H^+^-ATPase and the induction of MeJA, ABA, and [Ca^2+^]_cyt_ and subsequently promotes the elevation of ROS and NO. Apoplastic ROS production is mediated by BAK1, CPK5, and CBL1–CIPK11/26 complexes through RROH phosphorylation. On the one hand, apoplastic ROS activates GHR1 to mediate the activation of SLAC1 channels. On the other hand, apoplastic ROS activates Ca^2+^ channels by HPCA1. Elevated [Ca^2+^]_cyt_ activates sensor proteins, including CPKs, CBLs, and CIPKs. CDPK3/6, CBL5–CIPK11, and CBL1/9–CIPK23, which phosphorylate and activate SLAC1. CDPK21 and CDPK33 activate the K^+^_out_ channel GORK, whereas CDPK13 inhibits the K^+^_in_ channel KAT1 through phosphorylation. Arrows represent promotion. Black ended arrows represent suppression. P in the blue circle indicates phosphorylation. ABA, abscisic acid; CBLs, calcineurin B-likes; CIPK, CBL-interacting protein kinases; CPKs, Ca^2+^-dependent protein kinases; HPCA1, hydrogen peroxide-induced Ca^2+^ increases 1; GHR1, guard cell hydrogen peroxide-resistant 1; GORK, guard cell outward rectifying K^+^ channel; KAT1, K^+^ channel in *Arabidopsis thaliana* 1; MeJA, methyl jasmonate; NO, nitric oxide; PIP2;1, aquaporin; PM, plasma membrane; RBOH, respiratory burst oxidase homolog; ROS, reactive oxygen species; SLAC1, slow anion channel-associated 1.

**Table S1.** Host pH modulation by acidophilic and alkaliphilic pathogens.

| **Type** | **Pathogen** | **Host** | **Tissue** | **pH of healthy tissue** | **pH of infected tissue** | **ΔpH change** | **Time** | **Refs.** |
| --- | --- | --- | --- | --- | --- | --- | --- | --- |
| Acidophilic pathogen | *Penicillium digitatum* | Orange | Fruit | 4.77 | 3.12 | 1.65 | 7 d | [1] |
|  |  | Grapefruit | Fruit | 4.74 | 3.10 | 1.64 | 7 d | [1] |
|  | *Penicillium expansum* | Apple | Fruit | 4.31 | 3.88 | 0.43 | 7 d | [1] |
|  | *Penicillium italicum* | Orange | Fruit | 4.77 | 3.02 | 1.75 | 7 d | [1] |
|  |  | Grapefruit | Fruit | 4.55 | 3.23 | 1.32 | 7 d | [1] |
|  | *Phomopsis mangiferae* | Grape | Fruit | 3.80 | 2.50 | 1.30 | 7 d | [2] |
|  |  | Mango | Fruit | 5.10 | 4.10 | 1.00 | 7 d | [2] |
|  | *Sclerotinia sclerotiorum* | Apple | Leaf | 4.40 | 4.00 | 0.40 | 4 d | [3] |
|  |  | Carrot | Root | 6.00 | 3.00 | 3.00 | 4 d | [3] |
|  |  | Faba bean | Leaf | 5.37 | 3.51 | 1.86 | 3 d | [4] |
|  |  | Pea | Leaf | 5.58 | 3.96 | 1.62 | 3 d | [4] |
|  |  | Soybean | Leaf | 6.14 | 5.32 | 0.82 | 3 d | [4] |
|  | *Valsa mali* | Apple | Twig | 6.20 | 3.80 | 2.40 | 1 d | [5] |
| Alkaliphilic pathogen | *Alternaria alternata* | Cherry | Fruit | 4.30 | 6.10 | 1.80 | 7-10 d | [6] |
|  |  | Pepper | Fruit | 5.40 | 7.60 | 2.20 | 7-10 d | [7] |
|  |  | Persimmon | Fruit | 5.60 | 5.80 | 0.20 | 7-10 d | [7] |
|  |  | Melon | Fruit | 6.30 | 7.50 | 1.20 | 7-10 d | [7] |
|  |  | Tomato | Fruit | 5.60 | 7.40 | 1.80 | 7-10 d | [7] |
|  | *Blumeria graminis* | Barley | Leaf | 4.90 | 5.40 | 0.50 | 2 h | [8] |
|  | *Colletotrichum acutatum* | Apple | Fruit | 4.10 | 6.50 | 2.40 | 6-7 d | [9] |
|  | *Colletotrichum coccodes* | Apple | Fruit | 4.00 | 5.10 | 1.10 | 6-7 d | [9] |
|  |  | Tomato | Fruit | 5.50 | 8.00 | 2.50 | 5 d | [9] |
|  | *Colletotrichum gloeosporioides* | Avocado | Fruit | 6.50 | 7.00 | 0.50 | 7 d | [9] |
|  | *Fusarium graminearum* | Wheat | Leaf | 5.00 | 8.30 | 3.30 | 5 d | [10] |
|  | *Pseudomonas syringae* | Bean | Leaf | 4.80 | 5.10 | 0.30 | 5 h | [11] |

**References**

1. Prusky D, McEvoy JL, Saftner R, Conway WS, Jones R (2004) Relationship between host acidification and virulence of *Penicillium* spp. on apple and citrus fruit. Phytopathology 94(1):44–51. https://doi.org/10.1094/PHYTO.2004.94.1.44
2. Davidzon M, Alkan N, Kobiler I, Prusky D (2010) Acidification by gluconic acid of mango fruit tissue during colonization via stem end infection by *Phomopsis mangiferae*. Postharvest Biol Tec 55(2):71–77. https://doi.org/10.1016/j.postharvbio.2009.08.009
3. Billon-Grand G, Rascle C, Droux M, Rollins JA, Poussereau N (2012) pH modulation differs during sunflower cotyledon colonization by the two closely related necrotrophic fungi *Botrytis cinerea* and *Sclerotinia sclerotiorum*. Mol Plant Pathol 13(6):568–578. https://doi.org/10.1111/j.1364-3703.2011.00772.x
4. Xu L, Xiang M, White D, Chen W (2015) pH dependency of sclerotial development and pathogenicity revealed by using genetically defined oxalate‐minus mutants of *Sclerotinia sclerotiorum*. Environ. Microbiol 17(8):2896–2909. https://doi.org/10.1111/1462-2920.12818
5. Wu Y, Yin Z, Xu L, Feng H, Huang L (2018) VmPacC is required for acidification and virulence in *Valsa mali*. Front Microbiol 9:1981. https://doi.org/10.3389/fmicb.2018.01981
6. Prusky D, Yakoby N (2003) Pathogenic fungi: leading or led by ambient pH?. Mol Plant Pathol 4(6):509–516. https://doi.org/10.1046/J.1364-3703.2003.00196.X
7. Eshel D, Miyara I, Ailing T, Dinoor A, Prusky D (2002) pH regulates endoglucanase expression and virulence of *Alternaria alternata* in persimmon fruit. Mol Plant Microbe Interact 15(8):774–779. https://doi.org/10.1094/MPMI.2002.15.8.774
8. Felle HH, Herrmann A, Hanstein S, Huchelhoven R, Kogel KH (2004) Apoplastic pH signaling in barley leaves attacked by the powdery mildew fungus *Blumeria graminis* f. sp. hordei. Mol Plant Microbe Interact 17(1):118–123. https://doi.org/10.1094/MPMI.2004.17.1.118
9. Prusky D, McEvoy JL, Leverentz B, Conway WS (2001) Local modulation of host pH by *Colletotrichum* species as a mechanism to increase virulence. Mol Plant Microbe Interact 14(9):1105–1113. https://doi.org/10.1094/MPMI.2001.14.9.1105
10. Gu Q, Wang Y, Zhao X, Yuan B, Zhang M, Tan Z, Zhang X, Chen Y, Wu H, Luo Y, Keller NP, Gao X, Ma Z (2022) Inhibition of histone acetyltransferase GCN5 by a transcription factor FgPacC controls fungal adaption to host-derived iron stress. Nucleic Acids Res 50(11):6190–6210. https://doi.org/10.1093/nar/gkac498
11. O’Leary BM, Neale HC, Geilfus CM, Jackson RW, Arnold DL, Preston GM (2016) Early changes in apoplast composition associated with defence and disease in interactions between *Phaseolus vulgaris* and the halo blight pathogen *Pseudomonas syringae* pv. phaseolicola. Plant Cell Environ 39(10):2172–2184. https://doi.org/10.1111/pce.12770
